# Supplementary material for: Exploratory analysis of immunization records highlights decreased SARS-CoV-2 rates in individuals with recent non-COVID-19 vaccinations
Source: Sci Rep. 2021 Feb 26;11:4741. doi: 10.1038/s41598-021-83641-y (PMC7910541; doi:10.1038/s41598-021-83641-y)
Supplement: Supplementary file 1 — Supplementary Information. [file 41598_2021_83641_MOESM1_ESM.docx]

**Supplementary Material**

**Exploratory analysis of immunization records highlights decreased SARS-CoV-2 rates in individuals with recent non-COVID-19 vaccinations**

Colin Pawlowski^1+^, Arjun Puranik^1+^, Hari Bandi^1^, AJ Venkatakrishnan^1^, Vineet Agarwal^1^, Richard Kennedy^2^, John C. O’Horo^2^, Gregory J. Gores^2^, Amy W. Williams^2^, John Halamka^2^, Andrew D. Badley^2^, Venky Soundararajan^1^*

1. nference, inc., One Main Street, Suite 400, East Arcade, Cambridge, MA 02142, USA
2. Mayo Clinic, Rochester, MN, USA

- Joint first authors

* Address correspondence to VS (venky@nference.net)

**Figure S1: Age distributions in vaccinated (matched) and unvaccinated (matched) cohorts.**
For each vaccine associated with lower SARS-CoV-2 rates, age distributions for vaccinated (matched) and unvaccinated (matched) cohorts at the 1 year time horizon are shown. Vaccinated cohorts are shown in blue and unvaccinated cohorts are shown in orange. Numbers of patients in age ranges 0-9, 10-19, 20-29, 30-39, 40-49, 50-59, 60-69, 70-70, 80-80, and 90+ are shown for the following vaccines at the 1 year time horizon: **(A)** Geriatric Flu vaccine, **(B)** Pneumococcal Conjugate (PCV13), **(C)** Hepatitis A / Hepatitis B (HepA-HepB), **(D)** Measles-Mumps-Rubella (MMR), **(E)** Polio, **(F)** Haemophilus Influenzae type B (HIB), and **(G)** Varicella.


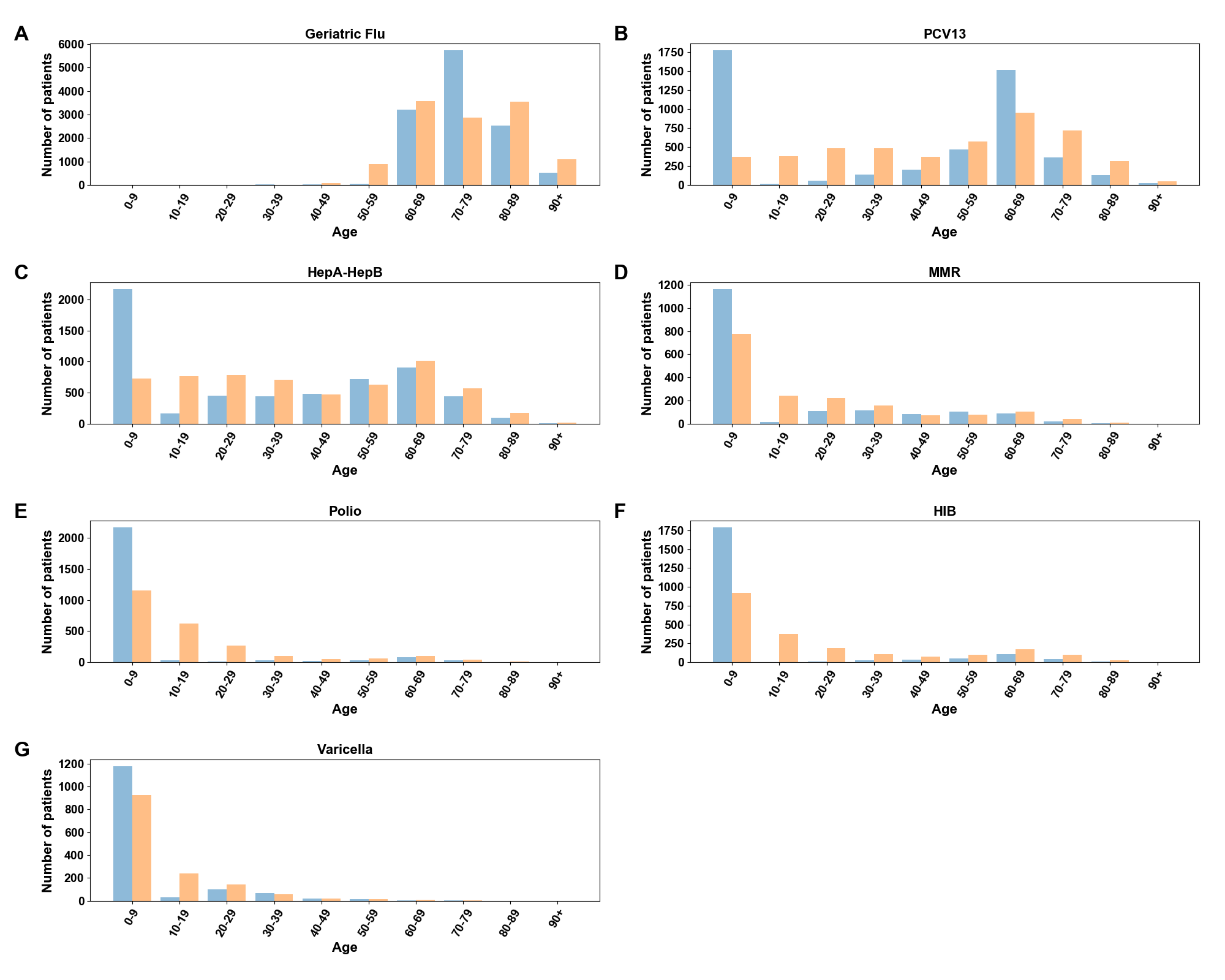


**Table S1. Covariate balance for Geriatric Flu vaccine over 1-year time horizon.** Mean/proportion values are shown for a selection of covariates for the vaccinated (matched), unvaccinated (matched), vaccinated (original), and unvaccinated (original) cohorts.

| **Covariate** | **Mean/**  **proportion among vaccinated (n=12085)** | **Mean/proportion among unvaccinated (n=12085)** | **Unmatched mean/proportion among vaccinated (n=13724)** | **Unmatched mean/proportion among unvaccinated (n=123313)** |
| --- | --- | --- | --- | --- |
| **COVIDpos rate** | **1.57%** | **2.13%** | 1.49% | 4.44% |
| County incidence | 0.12% | 0.13% | 0.12% | 0.14% |
| County PCR test positive rate | 4.48% | 4.74% | 4.45% | 5.20% |
| Age (std. dev) | 74.8 (7.7) | 74.5 (11.4) | 75.4 (7.9) | 47.0 (21.1) |
| Gender - Male | 5823 (48.2%) | 5855 (48.4%) | 6632 (48.3%) | 54080 (43.9%) |
| Race - White | 11590 (95.9%) | 11502 (95.2%) | 13173 (96%) | 106806 (86.6%) |
| Race - Black | 158 (1.31%) | 203 (1.68%) | 171 (1.25%) | 5302 (4.3%) |
| Race - Asian | 127 (1.05%) | 133 (1.1%) | 154 (1.12%) | 3113 (2.52%) |
| Ethnicity - Hispanic | 185 (1.53%) | 211 (1.75%) | 198 (1.44%) | 7522 (6.1%) |
| BMI (std. dev) | 29.2 (6.6) | 29.0 (6.6) | 29.2 (6.5) | 28.6 (7.6) |
| Elixhauser - Hypertension | 9216 (76.3%) | 9041 (74.8%) | 10672 (77.8%) | 37095 (30.1%) |
| Elixhauser - Pulmonary | 4414 (36.5%) | 4171 (34.5%) | 5125 (37.3%) | 25010 (20.3%) |
| Elixhauser - Diabetes mellitus | 989 (8.18%) | 981 (8.12%) | 1121 (8.17%) | 5486 (4.45%) |
| Elixhauser - Diabetes mellitus (complications) | 2580 (21.3%) | 2409 (19.9%) | 3014 (22%) | 8787 (7.13%) |
| Elixhauser - Coagulopathy | 1687 (14%) | 1650 (13.7%) | 1968 (14.3%) | 7166 (5.81%) |
| Elixhauser - Obesity | 4404 (36.4%) | 4181 (34.6%) | 5077 (37%) | 28257 (22.9%) |
| Pregnancy - 90 days preceding | 3 (0.0248%) | 2 (0.0165%) | 4 (0.0291%) | 2558 (2.07%) |
| # unique other vaccines taken over preceding 5y | 3.93 | 3.63 | 4.05 | 1.65 |
| Propensity score | 0.82 | 0.80 | 0.84 | 0.16 |

**Table S2. Covariate balance for Pneumococcal conjugate (PCV13) over 1-year time horizon.** Mean/proportion values are shown for a selection of covariates for the vaccinated (matched), unvaccinated (matched), vaccinated (original), and unvaccinated (original) cohorts.

| **Covariate** | **Mean/**  **proportion among vaccinated (n=4693)** | **Mean/proportion among unvaccinated (n=4693)** | **Unmatched mean/proportion among vaccinated (n=4693)** | **Unmatched mean/proportion among unvaccinated (n=132344)** |
| --- | --- | --- | --- | --- |
| **COVID_pos_ rate** | **2.17%** | **3.03%** | 2.17% | 4.21% |
| County incidence | 0.12% | 0.12% | 0.12% | 0.14% |
| County PCR test positive rate | 4.64% | 4.64% | 4.64% | 5.14% |
| Age (std. dev) | 38.7 (31.2) | 48.8 (24.4) | 38.7 (31.2) | 50.2 (21.4) |
| Gender - Male | 2404 (51.2%) | 2297 (48.9%) | 2404 (51.2%) | 58308 (44.1%) |
| Race - White | 4116 (87.7%) | 4192 (89.3%) | 4116 (87.7%) | 115863 (87.5%) |
| Race - Black | 197 (4.2%) | 170 (3.62%) | 197 (4.2%) | 5276 (3.99%) |
| Race - Asian | 108 (2.3%) | 102 (2.17%) | 108 (2.3%) | 3159 (2.39%) |
| Ethnicity - Hispanic | 301 (6.41%) | 222 (4.73%) | 301 (6.41%) | 7419 (5.61%) |
| BMI (std. dev) | 24.8 (8.0) | 28.0 (7.7) | 29.2 (6.5) | 28.6 (7.6) |
| Elixhauser - Hypertension | 1728 (36.8%) | 2237 (47.7%) | 1728 (36.8%) | 46039 (34.8%) |
| Elixhauser - Pulmonary | 1013 (21.6%) | 1390 (29.6%) | 1013 (21.6%) | 29122 (22%) |
| Elixhauser - Diabetes mellitus | 200 (4.26%) | 278 (5.92%) | 200 (4.26%) | 6407 (4.84%) |
| Elixhauser - Diabetes mellitus (complications) | 539 (11.5%) | 737 (15.7%) | 539 (11.5%) | 11262 (8.51%) |
| Elixhauser - Coagulopathy | 534 (11.4%) | 527 (11.2%) | 534 (11.4%) | 8600 (6.5%) |
| Elixhauser - Obesity | 1036 (22.1%) | 1404 (29.9%) | 1036 (22.1%) | 32298 (24.4%) |
| Pregnancy - 90 days preceding | 6 (0.128%) | 7 (0.149%) | 6 (0.128%) | 2556 (1.93%) |
| # unique other vaccines taken over preceding 5y | 4.37 | 4.74 | 4.37 | 1.79 |
| Propensity score | 0.66 | 0.66 | 0.66 | 0.34 |

**Table S3. Covariate balance for HepA-HepB over 1-year time horizon.** Mean/proportion values are shown for a selection of covariates for the vaccinated (matched), unvaccinated (matched), vaccinated (original), and unvaccinated (original) cohorts.

| **Covariate** | **Mean/**  **proportion among vaccinated (n=5858)** | **Mean/proportion among unvaccinated (n=5858)** | **Unmatched mean/proportion among vaccinated (n=5858)** | **Unmatched mean/proportion among unvaccinated (n=131179)** |
| --- | --- | --- | --- | --- |
| **COVID_pos_ rate** | **3.23%** | **4.01%** | 3.23% | 4.19% |
| County incidence | 0.12% | 0.12% | 0.12% | 0.14% |
| County PCR test positive rate | 4.58% | 4.62% | 4.58% | 5.15% |
| Age (std. dev) | 32.6 (28.0) | 40.5 (24.2) | 32.6 (28.0) | 50.6 (21.2) |
| Gender - Male | 2806 (47.9%) | 2600 (44.4%) | 2806 (47.9%) | 57906 (44.1%) |
| Race - White | 4936 (84.3%) | 5020 (85.7%) | 4936 (84.3%) | 115043 (87.7%) |
| Race - Black | 309 (5.27%) | 296 (5.05%) | 309 (5.27%) | 5164 (3.94%) |
| Race - Asian | 198 (3.38%) | 210 (3.58%) | 198 (3.38%) | 3069 (2.34%) |
| Ethnicity - Hispanic | 414 (7.07%) | 347 (5.92%) | 414 (7.07%) | 7306 (5.57%) |
| BMI (std. dev) | 25.2 (8.6) | 28.0 (7.7) | 25.2 (8.6) | 28.8 (7.4) |
| Elixhauser - Hypertension | 1839 (31.4%) | 2238 (38.2%) | 1839 (31.4%) | 45928 (35%) |
| Elixhauser - Pulmonary | 1227 (20.9%) | 1663 (28.4%) | 1227 (20.9%) | 28908 (22%) |
| Elixhauser - Diabetes mellitus | 285 (4.87%) | 372 (6.35%) | 285 (4.87%) | 6322 (4.82%) |
| Elixhauser - Diabetes mellitus (complications) | 891 (15.2%) | 1085 (18.5%) | 891 (15.2%) | 10910 (8.32%) |
| Elixhauser - Coagulopathy | 727 (12.4%) | 749 (12.8%) | 727 (12.4%) | 8407 (6.41%) |
| Elixhauser - Obesity | 1430 (24.4%) | 1931 (33%) | 1430 (24.4%) | 31904 (24.3%) |
| Pregnancy - 90 days preceding | 46 (0.785%) | 52 (0.888%) | 46 (0.785%) | 2516 (1.92%) |
| # unique other vaccines taken over preceding 5y | 4.90 | 5.43 | 4.90 | 1.81 |
| Propensity score | 0.73 | 0.73 | 0.73 | 0.27 |

**Table S4. Covariate balance for MMR over 1-year time horizon.** Mean/proportion values are shown for a selection of covariates for the vaccinated (matched), unvaccinated (matched), vaccinated (original), and unvaccinated (original) cohorts.

| **Covariate** | **Mean/proportion among vaccinated (n=1700)** | **Mean/proportion among unvaccinated (n=1700)** | **Unmatched mean/proportion among vaccinated (n=1737)** | **Unmatched mean/proportion among unvaccinated (n=135300)** |
| --- | --- | --- | --- | --- |
| **COVID_pos_ rate** | **3.12%** | **5.53%** | 3.05% | 4.16% |
| County incidence | 0.13% | 0.14% | 0.13% | 0.14% |
| County PCR test positive rate | 4.69% | 4.93% | 4.68% | 5.13% |
| Age (std. dev) | 15.5 (21.2) | 20.6 (21.3) | 15.3 (21.1) | 50.3 (21.5) |
| Gender - Male | 776 (45.6%) | 653 (38.4%) | 803 (46.2%) | 59909 (44.3%) |
| Race - White | 1431 (84.2%) | 1341 (78.9%) | 1462 (84.2%) | 118517 (87.6%) |
| Race - Black | 75 (4.41%) | 86 (5.06%) | 75 (4.32%) | 5398 (3.99%) |
| Race - Asian | 55 (3.24%) | 84 (4.94%) | 56 (3.22%) | 3211 (2.37%) |
| Ethnicity - Hispanic | 162 (9.53%) | 193 (11.4%) | 167 (9.61%) | 7553 (5.58%) |
| BMI (std. dev) | 20.7 (7.2) | 22.4 (7.8) | 20.6 (7.2) | 28.8 (7.5) |
| Elixhauser - Hypertension | 170 (10%) | 201 (11.8%) | 171 (9.84%) | 47596 (35.2%) |
| Elixhauser - Pulmonary | 249 (14.6%) | 287 (16.9%) | 251 (14.5%) | 29884 (22.1%) |
| Elixhauser - Diabetes mellitus | 24 (1.41%) | 38 (2.24%) | 24 (1.38%) | 6583 (4.87%) |
| Elixhauser - Diabetes mellitus (complications) | 47 (2.76%) | 57 (3.35%) | 47 (2.71%) | 11754 (8.69%) |
| Elixhauser - Coagulopathy | 64 (3.76%) | 71 (4.18%) | 64 (3.68%) | 9070 (6.7%) |
| Elixhauser - Obesity | 186 (10.9%) | 238 (14%) | 186 (10.7%) | 33148 (24.5%) |
| Pregnancy - 90 days preceding | 31 (1.82%) | 43 (2.53%) | 31 (1.78%) | 2531 (1.87%) |
| # unique other vaccines taken over preceding 5y | 6.61 | 6.95 | 6.72 | 1.93 |
| Propensity score | 0.80 | 0.80 | 0.80 | 0.20 |

**Table S5. Covariate balance for Polio over 1-year time horizon.** Mean/proportion values are shown for a selection of covariates for the vaccinated (matched), unvaccinated (matched), vaccinated (original), and unvaccinated (original) cohorts.

| **Covariate** | **Mean/proportion among vaccinated (n=2402)** | **Mean/proportion among unvaccinated (n=2402)** | **Unmatched mean/proportion among vaccinated (n=2440)** | **Unmatched mean/proportion among unvaccinated (n=134597)** |
| --- | --- | --- | --- | --- |
| **COVID_pos_ rate** | **2.66%** | **4.70%** | 2.62% | 4.17% |
| County incidence | 0.12% | 0.14% | 0.12% | 0.14% |
| County PCR test positive rate | 4.56% | 4.84% | 4.54% | 5.13% |
| Age (std. dev) | 6.5 (15.9) | 16.5 (17.9) | 6.5 (15.8) | 50.6 (21.1) |
| Gender - Male | 1313 (54.7%) | 1248 (52%) | 1339 (54.9%) | 59373 (44.1%) |
| Race - White | 1998 (83.2%) | 1894 (78.9%) | 2033 (83.3%) | 117946 (87.6%) |
| Race - Black | 116 (4.83%) | 150 (6.24%) | 117 (4.8%) | 5356 (3.98%) |
| Race - Asian | 74 (3.08%) | 114 (4.75%) | 74 (3.03%) | 3193 (2.37%) |
| Ethnicity - Hispanic | 228 (9.49%) | 196 (8.16%) | 229 (9.39%) | 7491 (5.57%) |
| BMI (std. dev) | 18.1 (3.9) | 20.7 (6.1) | 18.1 (3.9) | 28.9 (7.4) |
| Elixhauser - Hypertension | 118 (4.91%) | 362 (15.1%) | 120 (4.92%) | 47647 (35.4%) |
| Elixhauser - Pulmonary | 205 (8.53%) | 352 (14.7%) | 205 (8.4%) | 29930 (22.2%) |
| Elixhauser - Diabetes mellitus | 10 (0.416%) | 34 (1.42%) | 10 (0.41%) | 6597 (4.9%) |
| Elixhauser - Diabetes mellitus (complications) | 25 (1.04%) | 81 (3.37%) | 25 (1.02%) | 11776 (8.75%) |
| Elixhauser - Coagulopathy | 139 (5.79%) | 304 (12.7%) | 141 (5.78%) | 8993 (6.68%) |
| Elixhauser - Obesity | 82 (3.41%) | 205 (8.53%) | 82 (3.36%) | 33252 (24.7%) |
| Pregnancy - 90 days preceding | 2 (0.0833%) | 2 (0.0833%) | 2 (0.082%) | 2560 (1.9%) |
| # unique other vaccines taken over preceding 5y | 5.95 | 7.05 | 6.05 | 1.92 |
| Propensity score | 0.92 | 0.92 | 0.93 | 0.08 |

**Table S6. Covariate balance for HIB over 1-year time horizon.** Mean/proportion values are shown for a selection of covariates for the vaccinated (matched), unvaccinated (matched), vaccinated (original), and unvaccinated (original) cohorts.

| **Covariate** | **Mean/proportion among vaccinated (n=2061)** | **Mean/proportion among unvaccinated (n=2061)** | **Unmatched mean/proportion among vaccinated (n=2063)** | **Unmatched mean/proportion among unvaccinated (n=134974)** |
| --- | --- | --- | --- | --- |
| **COVID_pos_ rate** | **2.09%** | **3.93%** | 2.08% | 4.18% |
| County incidence | 0.11% | 0.13% | 0.11% | 0.14% |
| County PCR test positive rate | 4.44% | 4.85% | 4.44% | 5.13% |
| Age (std. dev) | 8.7 (20.4) | 22.8 (23.6) | 8.6 (20.4) | 50.5 (21.3) |
| Gender - Male | 1120 (54.3%) | 1056 (51.2%) | 1121 (54.3%) | 59591 (44.1%) |
| Race - White | 1743 (84.6%) | 1753 (85.1%) | 1744 (84.5%) | 118235 (87.6%) |
| Race - Black | 87 (4.22%) | 78 (3.78%) | 87 (4.22%) | 5386 (3.99%) |
| Race - Asian | 57 (2.77%) | 66 (3.2%) | 57 (2.76%) | 3210 (2.38%) |
| Ethnicity - Hispanic | 188 (9.12%) | 175 (8.49%) | 188 (9.11%) | 7532 (5.58%) |
| BMI (std. dev) | 18.7 (4.4) | 21.9 (7.3) | 18.7 (4.4) | 28.8 (7.4) |
| Elixhauser - Hypertension | 176 (8.54%) | 512 (24.8%) | 178 (8.63%) | 47589 (35.3%) |
| Elixhauser - Pulmonary | 168 (8.15%) | 386 (18.7%) | 168 (8.14%) | 29967 (22.2%) |
| Elixhauser - Diabetes mellitus | 17 (0.825%) | 65 (3.15%) | 17 (0.824%) | 6590 (4.88%) |
| Elixhauser - Diabetes mellitus (complications) | 53 (2.57%) | 188 (9.12%) | 53 (2.57%) | 11748 (8.7%) |
| Elixhauser - Coagulopathy | 168 (8.15%) | 362 (17.6%) | 170 (8.24%) | 8964 (6.64%) |
| Elixhauser - Obesity | 85 (4.12%) | 285 (13.8%) | 86 (4.17%) | 33248 (24.6%) |
| Pregnancy - 90 days preceding | 1 (0.0485%) | 1 (0.0485%) | 1 (0.0485%) | 2561 (1.9%) |
| # unique other vaccines taken over preceding 5y | 5.10 | 6.50 | 5.11 | 1.95 |
| Propensity score | 0.90 | 0.89 | 0.90 | 0.11 |

**Table S7. Covariate balance for Varicella over 1-year time horizon.** Mean/proportion values are shown for a selection of covariates for the vaccinated (matched), unvaccinated (matched), vaccinated (original), and unvaccinated (original) cohorts.

| **Covariate** | **Mean/**  **proportion among vaccinated (n=1416)** | **Mean/proportion among unvaccinated (n=1416)** | **Unmatched mean/proportion among vaccinated (n=1458)** | **Unmatched mean/proportion among unvaccinated (n=135579)** |
| --- | --- | --- | --- | --- |
| **COVID_pos_ rate** | **2.75%** | **4.45%** | 2.88% | 4.16% |
| County incidence | 0.13% | 0.14% | 0.12% | 0.14% |
| County PCR test positive rate | 4.59% | 4.98% | 4.58% | 5.13% |
| Age (std. dev) | 7.3 (11.9) | 10.2 (12.1) | 7.2 (11.7) | 50.3 (21.5) |
| Gender - Male | 696 (49.2%) | 544 (38.4%) | 699 (47.9%) | 60013 (44.3%) |
| Race - White | 1203 (85%) | 1087 (76.8%) | 1239 (85%) | 118740 (87.6%) |
| Race - Black | 54 (3.81%) | 69 (4.87%) | 54 (3.7%) | 5419 (4%) |
| Race - Asian | 48 (3.39%) | 97 (6.85%) | 51 (3.5%) | 3216 (2.37%) |
| Ethnicity - Hispanic | 137 (9.68%) | 176 (12.4%) | 141 (9.67%) | 7579 (5.59%) |
| BMI (std. dev) | 18.8 (5.7) | 20.0 (6.2) | 18.8 (5.6) | 28.8 (7.5) |
| Elixhauser - Hypertension | 37 (2.61%) | 32 (2.26%) | 37 (2.54%) | 47730 (35.2%) |
| Elixhauser - Pulmonary | 159 (11.2%) | 146 (10.3%) | 161 (11%) | 29974 (22.1%) |
| Elixhauser - Diabetes mellitus | 6 (0.424%) | 8 (0.565%) | 6 (0.412%) | 6601 (4.87%) |
| Elixhauser - Diabetes mellitus (complications) | 10 (0.706%) | 12 (0.847%) | 10 (0.686%) | 11791 (8.7%) |
| Elixhauser - Coagulopathy | 38 (2.68%) | 43 (3.04%) | 38 (2.61%) | 9096 (6.71%) |
| Elixhauser - Obesity | 76 (5.37%) | 124 (8.76%) | 77 (5.28%) | 33257 (24.5%) |
| Pregnancy - 90 days preceding | 12 (0.847%) | 21 (1.48%) | 12 (0.823%) | 2550 (1.88%) |
| # unique other vaccines taken over preceding 5y | 6.98 | 7.12 | 7.12 | 1.94 |
| Propensity score | 0.88 | 0.88 | 0.89 | 0.11 |
